# Multimodal Integration and Phenomenal Spatiotemporal Binding: A Perspective From the Default Space Theory

**DOI:** 10.3389/fnint.2019.00002

**Published:** 2019-02-05

**Authors:** Ravinder Jerath, Connor Beveridge

**Affiliations:** Charitable Medical Healthcare Foundation, Augusta, GA, United States

**Keywords:** default space, binding problem, metastable, oscillations, consciousness, phenomenology, multimodal integration, neural synchronization

## Abstract

How does the integrated and unified conscious experience arise from the vastly distributed activities of the nervous system? How is the information from the many cones of the retina bound with information coming from the cochlea to create the association of sounds with objects in visual space? In this perspective article, we assert a novel viewpoint on the “binding problem” in which we explain a metastable operation of the brain and body that may provide insight into this problem. In our view which is a component of the Default Space Theory (DST), consciousness arises from a metastable synchronization of local computations into a global coherence by a framework of widespread slow and ultraslow oscillations coordinated by the thalamus. We reinforce a notion shared by some consciousness researchers such as Revonsuo and the Fingelkurts that a spatiotemporal matrix is the foundation of phenomenological experience and that this phenomenology is directly tied to bioelectric operations of the nervous system. Through the oscillatory binding system we describe, cognitive neuroscientists may be able to more accurately correlate bioelectric activity of the brain and body with the phenomenology of human experience.

## Introduction

Phenomenology is the reflection on and analysis of the essential structure and form of conscious experience (Husserl, 1952; Menon et al., 2013). An understanding of the structure and dynamics of the phenomenology of consciousness offers constraints on the search for its explanatory mechanisms in the brain and potentially the body (Revonsuo, 2003). In this article, we assert a viewpoint on not only the phenomenology, but the physiological underpinnings of consciousness which may bridge the gap in understanding the connection between the material and experiential components of it. We propound that a subconscious, virtual, space-time matrix is the foundation of animal experience and continuously exists in the conscious mind as a coordinate system for a recreation or simulation of the material world. Additionally, this matrix is physically created by a global, unified, bioelectric oscillatory structure that spans the brain and body.

The Default Space Theory (DST; Jerath et al., 2015; Jerath and Beveridge, 2018) is a novel, metastable, embodied theory of consciousness which is unique in its holistic description of the body as an integral component of perception. Metastable models such as the Operational Architectonics Theory (Fingelkurts and Fingelkurts, 2001; Fingelkurts et al., 2010, 2013) and Global Workspace Theory (Edelman et al., 2011) show promise in revealing the nature of consciousness. Theories of metastability propose that consciousness arises from the global integration and coordination of distinct mesoscopic neural modules that while perform their own innate functions, couple together to form a large-scale coherence (Freeman and Holmes, 2005; Kelso and Tognoli, 2007; Fingelkurts and Fingelkurts, 2017). The DST extends this metastable architecture to the sensory receptors themselves (Jerath and Beveridge, 2018), thus extending the science of embodied cognition into consciousness. Embodied cognition is a radical field in that diverts from the prevailing view that cognition is a sole processes of the brain by asserting the body plays a crucial role in our psychology (Foglia and Wilson, 2013).

There still exists a debate as to whether neural oscillations play a functional role in cognition and consciousness, if they arise simply as a epiphenomenal byproduct of spiking activity, or even if they interfere with normal processing (Koepsell et al., 2010; Chalk et al., 2016). We share the prevailing perspective that bioelectric oscillations play a variety of key roles in neural processes responsible for cognition (Fries, 2005, 2009; Cole and Voytek, 2017; Wutz et al., 2018) as well as the unified nature of consciousness (Fingelkurts et al., 2010). Increasing evidence supporting this perspective reveal that neural oscillations are a powerful means to transfer and encode information (Cheong and Levchenko, 2010). Oscillations may provide a necessary low-energy mechanism for local and distant communication lost in mere action potential signaling (Buzsáki and Draguhn, 2004) which in larger brains would have severe spatial and metabolic constraints (Knyazev, 2012). The fact that the full frequency spectrum of oscillations is phylogenetically preserved strongly suggests they serve important functional purposes (Buzsáki and Draguhn, 2004).

## The Binding Problem

The binding problem is a considerable mystery in cognitive science which ponders how a unified experience could arise from the distributed and disparate activities of the nervous system (Revonsuo and Newman, 1999). There are many aspects of binding including property, part, location, and temporal binding and sub-problems such as how certain stimuli are bound to discrete objects in perceptual space (Revonsuo and Newman, 1999). Perceptual representations depend on neural codes that constitute the parts and properties of physical objects perceptually recreated (Treisman, 1996). Due to the fact that only a fraction of all neural processing enters consciousness, there must be some mechanism for dynamic selection of neural assemblies that enter awareness (Engel and Singer, 2001). We support the well-supported notion that achieving such selection along with cross-modal coherence requires mechanisms for binding neural information (Singer et al., 1997). An effective theory on multisensory integration must answer at least two pivotal problems: how is information integrated across distal cortical and subcortical regions, and how are unrelated signals within the same processing modules segregated. We believe an underlying oscillatory architecture coordinated by the thalamus binds and segregates such streams of activity based on the spatial and temporal locations of each stimulus in external and thus phenomenal space.

The temporal binding model proposes neurons responding to identical sensory objects or scenes in space synchronize in the millisecond range while this synchronization does not exist between neurons representing separate objects in external space (Singer and Gray, 1995; Engel and Singer, 2001). We further this concept by suggesting the sensory receptors themselves are synchronized with associated cortical areas. Consciousness has been argued to result from thalamocortical circuits which bind sensory contents encoded by thalamocortical loops (Llinás and Ribary, 1994) which become globally available (Baars et al., 2013), and we stress the important of the thalamus in global binding. Many authors have proposed that electrical thalamocortical coherence is the functional basis for binding (Llinás et al., 1998, 2002) and that coupled oscillatory activity may serve to link simultaneous neural activity across multisensory regions (Senkowski et al., 2008). We support this perspective and describe a global, underlying oscillatory structure into which all activity potentially producing sensory qualia are dynamically bound.

## A Phenomenological Space-Time Matrix

**Figure 1:**
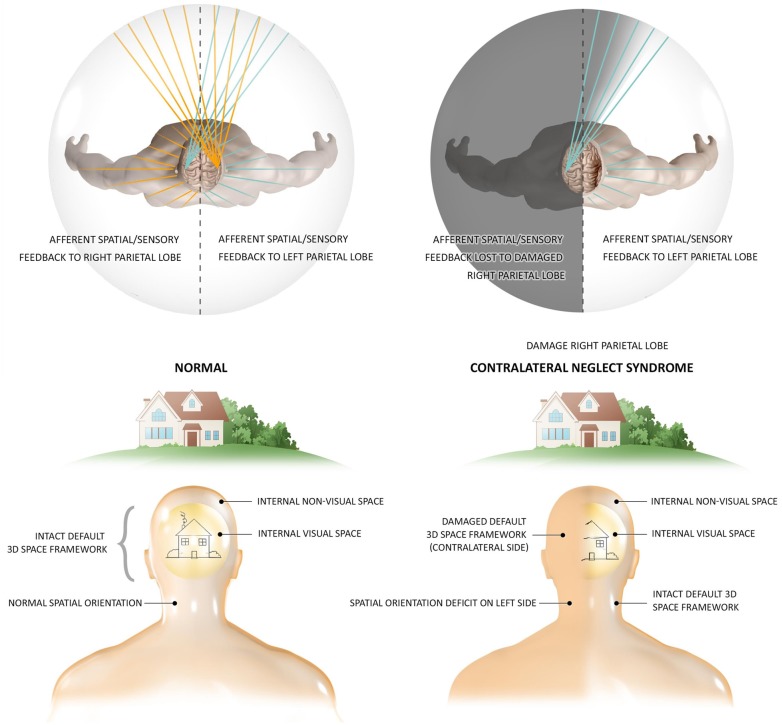
The existence of an essential phenomenal space revealed by contralateral neglect syndrome. Contralateral Neglect is a condition resulting often from damage the right parietal lobe (Kerkhoff, 2001). Not only do the neglected side of space and all sensation within it disappear from consciousness in this condition, but these patients are not aware of any “missing” space and experience the right side as the full external world. They may eat from one side of their plate or dress one side of their body. Two main theories exist at as why this may occur, both supporting the existence of an unconscious virtual coordinate matrix as the basis of consciousness. In the first theory, as illustrated in the top image, the parietal brain area maps percepts spatially. The right lobe maps both sides of the perceptual field while the left only one side, thus, lesions of the right side lead to a lack of mapping of the left perceptual field (Iachini et al., 2009). The necessity of spatial mapping of percepts into a 3D coordinate matrix for experience is revealed by the loss of perception of the entire left side of space for these patients (Jerath and Crawford, 2014), illustrated by the bottom image. Recent findings have stimulated the second theory which focuses on disturbed resting brain oscillation networks of Alpha frequency is supported by the fact that neglect can occur from damage to numerous right-side cortical and sub-cortical areas (Corbetta and Shulman, 2011), and that unconscious processing of the neglected stimuli still occur. In this theory, lesions damage these networks which are responsible for spatial attention. This supports our view that Alpha coherence brings the unconscious virtual space to awareness and that a spatial component to qualia must exist for it to enter consciousness (figure by Lynsey Ekema, MSMI). Previously Published in Jerath and Beveridge (2018). Permission obtained by Creative Commons.

In our perspective shared by a subset of consciousness researchers, the most fundamental aspect of human experience is a subconscious (Damasio, 1999), unifying, empty, spatial coordinate matrix in which all qualia must be embedded in order to come into conscious awareness. In everyone’s experience, we perceive the world from the center or mathematical origin of this externalized space (Revonsuo, 2006). This center provides a spatial sense of first-person self (Metzinger, 2003; Trehub, 2007; Blanke and Metzinger, 2009). This concept of a phenomenological space-time has been supported by neurophysiological and cognitive research (Weiskrantz, 1997), such as investigation into contralateral neglect syndrome (Driver and Vuilleumier, 2001; Figure 1), as well as our own everyday personal experience, and has been most thoroughly explored by the Fingelkurts in their Operational Architectonics Theory (Fingelkurts et al., 2010). Revonsuo has termed this space “virtual space,” and identified it as responsible for the global unity of consciousness (Revonsuo, 2006). Tononi et al. (2016) has described structure as an axiom of all consciousness, not just human.

We share the view that consciousness is an emergent phenomenon resulting from a functional representation or simulation of the external world and that the ontology of our phenomenological space-time is a direct replication of the dimensional nature of the physical universe (Siegel, 2006). This allows us the survival benefit of interacting optimally with our environment. Although phenomenal contents may be representations of the external world, they are never experienced as such, instead giving the impression that they are actual objects or scenes in the physical world (Metzinger, 2003). Support is given by the nature of dreams which reveal that experiences outside of perception of the external world are spatially structured (Strauch and Meier, 1996) and experienced as real the majority of the time (Farthing, 1992).

## An Underlying Cognitive Architecture

While there is significant support for a phenomenological space-time coordinate matrix as a foundation of experience, not much research has been invested in how this is reflected neurophysiologically. Our novel interpretation of this bridge between the material and experiential may provide great insight into binding and multisensory integration. This interpretation includes the notion that this subconscious space-time matrix is isomorphic to an ongoing, global, dynamic architecture of harmonious oscillatory activity (Jerath and Beveridge, 2018) upon which activity generating percepts build and bind upon (Freeman and Vitiello, 2006; Fingelkurts et al., 2010, 2013). In our view, the bioelectric structure responsible for this matrix is the most basic and important layer of a greater cognitive architecture spanning the entire body which produces consciousness. We assert that this phenomenal space and its isomorphic bioelectric structure are crucial to solving the binding problem as the objects and scenes upon which various modes of stimuli are bound (Singer and Gray, 1995) are in turn bound to this greater coordinate matrix.

We propose this underlying, global, operational structure provides a coherence mechanism for all sensory modalities to unify based upon their spatial coordinates in external space and may provide an explanation for baseline neural activity. Baseline activity was traditionally considered noise (Emadi et al., 2014), but has been realized at minimum to play an important role in perception and behavior (Supèr et al., 2003). Its incessant activity utilizes the majority of the brain’s energy (Raichle and Gusnard, 2002), but its exact function is still mysterious (Balduzzi et al., 2008). Ultraslow (<0.1 Hz) oscillations are known to be an intrinsic component of brain activity (Birn et al., 2006; Raichle and Snyder, 2007). Baseline activity has been found to interact with activity induced by an external stimulus in creating the overall response (Fox et al., 2005; Liu et al., 2011). According to our view, this subconscious, baseline, oscillatory layer of oscillations in neuronal membrane potential as well as at the macroscopic level operate at an ultraslow frequency while higher frequency oscillations responsible for qualia are bound to its virtual coordinate matrix *via* synchronization. Other theories of consciousness have also correlated higher frequency activity with consciousness and lower frequency with unconsciousness such as the Dynamic Core Hypothesis (Murphy and Brown, 2007).

Our perspective that the global cognitive architecture consists of multiple oscillatory layers (Jerath and Crawford, 2015) and that the most fundamental layer is global and responsible for producing the phenomenal coordinate matrix is supported by significant research. Oscillatory patterns have been shown to be hierarchically organized by frequency with the lower frequency activity underlying and correlating with high frequency activity (Monto et al., 2008; Yuan et al., 2012). In addition this lower frequency activity has been demonstrated to modulate (Lakatos et al., 2005), group (Steriade et al., 2001; Vanhatalo et al., 2004), organize, and entrain higher frequencies (Herrero et al., 2018) as well as be effective in facilitating long-range communication (Hyafil et al., 2015). We assert the fundamental low frequency layer is in part maintained and coordinated by cardiorespiratory activity (Tong et al., 2013; Heck et al., 2017; Varga and Heck, 2017; Herrero et al., 2018) and the modules of the Default Mode Network (Buckner et al., 2008; Fingelkurts et al., 2016). This coordination of the lowest layer provides a mechanism for global entrainment and harmony needed to create a unified and integrated experience.

According to most perspectives in phenomenology, qualia must have structure, composed of several experiential distinctions such as visual color, a location, or sound (Husserl, 1952; Brown, 2005; Menon et al., 2013; Oizumi et al., 2014). The lowest layer of ultra-slow oscillations creating the virtual coordinate matrix provides the global foundation for qualia to be built and bound both electrophysiologically and phenomenally. We believe the substance of frameworks for the diverse sensory modalities that fill this space in various forms consist largely of circuits bound by Alpha oscillations which provide the next level of structure required for qualia emerge. Just as the virtual matrix, sensory frameworks are continuously active in the awake state even with a lack of stimuli which is revealed by the presence of Alpha baseline activity (Iemi et al., 2017). We propose these frameworks in their empty form are significantly more conscious than the 3D matrix upon which they are bound, commonly demonstrated by the experience of complete darkness. Continentally blind people do not experience darkness as someone with eyes closed would; however, they have a complete lack of visual experience all together (Bryan Magee, 1995; Koster-Hale et al., 2014). Thus, we hold that while sensory frameworks are created through dynamic neural circuits and oscillatory networks, the type of sensation it creates is dependent upon the processing mechanisms of the system.

Synchrony of Alpha oscillations in addition may mediate the rise of these frameworks into conscious awareness *via* spatial attention (Sasaki et al., 2013; Fingelkurts and Fingelkurts, 2015). Recent findings on the diversity of brain areas that when damaged lead to the condition of contralateral neglect we mentioned suggests structural damage to specific brain areas may not significantly explain the condition. Instead, disruptions of resting Alpha networks that control spatial attention *via* inter-hemispheric connectivity provide a more sound explanation (Corbetta, 2012; Sasaki et al., 2013). We interpret these findings as evidence for our view that in addition to playing a major role in the formulation of sensory frameworks, Alpha rhythms bring the unconscious virtual space into awareness through further baseline functional connectivity needed for large-scale integration of local computations, and that spatial awareness is a fundamental prerequisite to any other type of awareness.

## Multimodal Integration

Imagine yourself along a busy street while you attempt to cross. Auditory and visual stimuli from passing cars provide reciprocal information on passing cars allowing you to safely cross, however these distinct categories of information must be integrated and unified for effective perception of crossing safety. Research has shown that different sensory modalities are indeed spatially and temporally integrated so that different qualities belonging to the same object are registered in the same space and time (Treisman, 1999; Watt and Phillips, 2000). Although it is still not well understood how this multisensory integration occurs, coherence in oscillatory activity is thought to be an essential mechanism (Fingelkurts et al., 2003; Senkowski et al., 2008). In the perspective we put forth here, distinct sensory frameworks substantiated by oscillatory systems of lower frequency such as alpha are integrated *via* the base oscillatory layer ultra-slow oscillations responsible for the spatial coordinate matrix we have described. The integration is primarily accomplished by binding and entrainment through similarity in space and time (Molholm et al., 2002; Talsma and Woldorff, 2005), which is facilitated by such a basal, oscillatory space-time structure. We also hold the opinion that qualia are integrated into the sensory frameworks *via* synchrony and filled into their respective phenomenological frameworks which are themselves filled into the 3D coordinate virtual matrix.

The dynamic nature and functional importance of audiovisual integration is demonstrated in a pioneering study showing incongruent auditory-visual speech signals result in a fused speech percept (Mcgurk and Macdonald, 1976). Responses to cross-modal stimuli are superior to uni-modal stimuli (Wang et al., 2013). The multitude of benefits from the combination of sensory modalities includes improved orientation (Stein et al., 1989), target detection (Frassinetti et al., 2002; Lovelace et al., 2003), and response times (Amlôt et al., 2003; Diederich et al., 2003). A large fraction of cortical activities are indeed formed from inputs of multiple sensory types, even the early, primary sensory sites (Ghazanfar and Schroeder, 2006; Kayser and Logothetis, 2007). Support for the modern hypothesis that dynamic, coherent oscillations play a key role in multisensory integration and in the selection of sensory information that matches across different sensory modalities has gained significant support in recent years (Fingelkurts et al., 2003; Herrmann et al., 2004; Fries, 2005). Bioelectric coherence provides a mechanism for specific patterns of functionally connectivity which would be required for the sensory frameworks we describe to hold.

Gamma band activity is shown to increase when auditory and visual signals are presented close in time (~25 ms; Senkowski et al., 2007). Greater Gamma band activity was also seen in audiovisual processing when semantically congruent stimuli were presented vs. incongruent stimuli (image of dog with a bark vs. a meow; Yuval-Greenberg and Deouell, 2007). In general, audiovisual Gamma activity appears to be enhanced when the auditory stimulus matches some visual pattern (Widmann et al., 2007). All levels of multisensory interactions are characterized by oscillatory responses most often in this Gamma band (Senkowski et al., 2008). This activity may reflect the formation of a unitary event representation (Widmann et al., 2007). In our perspective, higher frequency activity such as Gamma represent higher order qualia which are bound to subconscious frameworks which create large-scale networks needed for an integrated experience. The tightly localized activity of high frequency activity coordinate computations of specific sites while the global integration of these computations are achieved by the widespread slower oscillations (Singer, 2011).

In our view, the thalamus and the thalamocortical oscillations are additional key coordinators of oscillatory activity among the cortex and among the cortex and sensory receptors [our novel proposition (Jerath et al., 2016)], serving to coordinate the binding of multi-modal qualia to the sensory frameworks and these frameworks to the underlying virtual space. In this way, expectations may directly modulate the activity of sensory receptors (Jerath and Beveridge, 2018). There is increasing evidence that the thalamus may integrate different sensory stimuli and influence cortical multimodal processing *via* corticothalamic connections (Tyll et al., 2011). The sensory specific nuclei of the thalamus have also been evidenced to integrate different modalities and feed this multisensory information to primary sensory specific-cortices (Noesselt et al., 2007; Kayser et al., 2008). As the thalamus has vast reentrant and resonant connections with the cortex (Jones, 2007), it is a prime candidate for an integrator of diverse information across widely dispersed cortical sites (Cappe et al., 2012) providing a means of large-scale, simultaneous synchronized events which may conjoin in time all sensory inputs (Llinás et al., 2002). We assert the thalamus in tandem with the ultra-slow oscillations provide the means for metastable operations of binding and global unity of neural activity to occur by coordinating the integration of the vast and varied mental operations creating qualia into the virtual 3D matrix.

## Conclusions

In asserting our perspective on phenomenal space-time and its isomorphic bioelectric structure, we have endeavored to provide insight into the binding problem regarding multimodal integration. We have focused on audiovisual integration in this article to provide an important instance of how this underlying oscillatory structure may entrain and organize multisensory streams into a unified whole resulting in the integrated experience. We have put forth a hierarchical view on oscillatory cognitive frameworks which include a basal, unconscious virtual coordinate matrix characterized by slow oscillations and the subconscious sensory frameworks characterized largely by Alpha oscillations. The sensory frameworks are bound to the space-time framework upon which qualia are further bound represented by even higher frequency oscillations. This hierarchy is supported by research revealing functional baseline activity and the fact that slow oscillations underlie and entrain faster ones. By describing how the slow oscillations produce a virtual space-time matrix, we describe a means for disparate sensory modalities to be integrated. In addition to empirically identifying a global oscillatory framework underlying sensory frameworks, further research should investigate the role of different frequencies in multisensory integration.

## Author Contributions

Theory developed by RJ with some writing with majority of the manuscript written by CB.

## Conflict of Interest Statement

The authors declare that the research was conducted in the absence of any commercial or financial relationships that could be construed as a potential conflict of interest.
